# Social–ecological timelines to explore human adaptation to coastal change

**DOI:** 10.1007/s13280-018-1129-5

**Published:** 2018-12-19

**Authors:** Camilla Brattland, Einar Eythórsson, Jørn Weines, Knut Sunnanå

**Affiliations:** 1 UiT – the Arctic University of Norway, Tromsø, Norway; 2 Norwegian Institute for Cultural Heritage Research (NIKU), Oslo, Norway; 3 Institute of Marine Research, Bergen, Norway

**Keywords:** Alien invasive species, Indigenous and rural communities, Northern Norway, Social–ecological systems, Social–ecological timelines

## Abstract

Through the construction of a socio-ecological timeline for the Porsanger fjord ecosystem, this article illustrates the different ways in which environmental and social–ecological changes have influenced the adaptations of rural households in coastal Sami communities in Finnmark, north Norway. The main finding is that, although environmental change in the form of seal invasions and dwindling fish stocks directly impacted the fisheries, the introduction of a new vessel quota system decisively changed adaptive capacity and coastal Sami household adaptation strategies. These changes represented a tipping point for the social–ecological system in the period between 1986 and 1990. It is thus important to discuss the ways in which governance systems may facilitate actions to adapt to climate and biodiversity change and foster sustainable rural livelihood systems in coastal Norway. Based on traditional and local ecological knowledge on the state of the ecosystem prior to the tipping point, two relevant actions to increase the resilience of the system were identified: ensuring the possibility of re-entry into fisheries as part of rural livelihood combinations, and ecological restoration of kelp beds. Flexible diversification of livelihoods allows exploitation of a range of adjacent species without large investments in a fossile fuel-driven fisheries economy. Investing in regrowth of macroalgae to foster cod nursery areas and increase carbon sequestration can be a relevant alternative for communities that are interested in contributing to climate change mitigation on a larger scale.

## Introduction

How should rural and indigenous communities take action to maintain their traditional livelihood adaptations and social–ecological resilience in the face of climate and biodiversity change? In the context of coastal communities, Perry et al. (2011) point to diversification, reliance on subsistence self-employment, and seeking employment in other sectors as the traditional way to cope with poor fishing seasons. In the rural coastal communities of northern Norway, flexible mixed-economy household strategies with the opportunity to combine a range of available livelihoods is a key trait of indigenous and local adaptations to ecological change (i.e. Nilsen 1998). These strategies are, however, being challenged by a changing environment and by management policies that restrict access to fisheries based on a certain level of activity which are hard for many small-scale fishermen to keep up with. Discussing the resilience of coastal communities, Broderstad and Eythórsson (2014) point to political participation, and financial and political support mechanisms as key to the successful adaptation of the coastal Sami communities to changing social–ecological conditions over the past decades. In the social–ecological history of the Porsanger fjord, the most recent change is the introduction of the king crab (*Paralithodes camtschaticus*) fishery, which is an alien invasive species (IAS) introduced to Norwegian fjords from the late 1990s. The king crab is currently managed both as an invasive and a commercial species, with an increasingly important role in the mix of livelihoods in coastal communities. Referring to the positive influence of the commercial king crab fishery on local economies in Finnmark, Broderstad and Eythórsson conclude that “odd as it may seem, an irreversible change in the ecosystem has contributed positively to the reorganization and resilience of the social–ecological system” (ibid.). The main mechanism for this positive reorganization was a policy that favored the participation of small-scale fishermen in the king crab fishery and allowed for incorporation of a king crab quota into the already existing vessel quota system that was introduced in 1990. In 2015 however, changes to the king crab management system restricted the fishery to vessels above 6 m of length out of animal health concerns, thus closing out around 50 fishermen in Eastern Finnmark from the fishery if they did not invest in larger vessels (NRK 2014). This small example illustrates some of the ongoing adaptation challenges for rural households that do not necessarily want to invest in a fisheries economy with little room for economic diversification. In the current context of climate change, focusing on long-term adaptations and actions that may serve to lower greenhouse gas emissions and to foster livelihoods that increases the resilience of social–ecological systems is a central concern. What adaptation strategies and actions could then be facilitated? Howard (2013) argues that research on human adaptation to climate and biodiversity change should focus on rural subsistence societies and “on the ways in which these population groups autochthonously adapt or mal-adapt to biodiversity change” (Howard 2013, p. 16). Moreover, one exemplary focus should be on the way rural subsistence societies and their social–ecological systems adapt to IAS, especially since there is a dearth of research on impacts of IAS on local level benefits and trade-offs (ibid.). In response to this approach, through the construction of a social–ecological timeline of change and responses to change in the Porsanger fjord community, in the following we discuss past and ongoing adaptation strategies in coastal Sami communities to identify adaptations and actions that could meet the challenges posed by changing social–ecological conditions.

## Study area and methods

This paper is the result of a failed attempt to integrate the local ecological knowledge (LEK) of Sami fjord fishermen in marine science and management of a fjord ecosystem in Finnmark, northern Norway. In northern Norway, rural and indigenous coastal communities are dependent on the marine ecosystem and its ecosystem services, where the cod fishery is the single most important to small-scale and indigenous fishermen. The Porsanger municipality consists of a population of 3981 inhabitants (SSB 2018) spread out in small settlements along the shoreline with the main bulk of the population in the central village of Lakselv. Fisheries used to be one of the main livelihoods in combination with small-scale farming and part-time occupations such as the construction industry, in public services or teaching, while it is currently the smallest (from 120 registered fishermen in 1987 to only 17 in 2017). Fjord ecosystems in Finnmark are perhaps more accurately described as part of the open Barents Sea marine ecosystem neighboring the fjord system, and thus always influenced by larger-scale fluctuations originating from outside the fjord (Jakobsen and Ozhigin 2011). The long and wide fjord is divided in two by a shelf, with warmer, Atlantic water conditions characterizing the outer parts, while low temperatures of a polar character dominate the inner part (Pedersen et al. 2018). Due to lack of data on local temperature variations, local ecosystem variability has been seen in relation to changes in ocean temperature in the North Atlantic Ocean from the 1940s onwards. Fisheries on several local coastal cod stocks were conducted along the whole length of the fjord, but the fish resources in the innermost part increasingly dwindled with declining sea temperatures and increasing fishing pressure from the end of the 1970s.

In the mid-1980s, what became known as the Coastal Sami Uprising took place in the Porsanger fjord in Finnmark, as an indigenous Sámi protest against the increasing overexploitation of local cod stocks by fishing vessels using more effective fishing technology than the locals at the time (Nilsen 2003; Eythórsson 2008). Followed by a dramatic invasion of harp seals to the Finnmark coast (Nilssen and Haug 1995), the collapse of the local cod fisheries, and other ecological changes, the Porsanger municipality government invited marine scientists to investigate what could be done to bring the fjord ecosystem “back to life” (Søderholm 2002). Importantly, marine science and fisheries authorities were at the time heavily criticized for ignoring fishermen’s traditional and local ecological knowledge, as the impact of the ecological changes on local fisheries had not been taken into account in the distribution of individual vessel quotas (IVQ) that was introduced in 1990. Up until 1995, biological data for fjord systems were not systematically gathered, which resulted in a gap between fishermen’s ecological knowledge and marine science on the fjord ecosystem. However, through various research projects, the Institute of Marine Research initiated local acoustic trawl surveys of the fjord, first in 1992 and then annually from 1995 as part of the marine research cruises. Sporadically at first, and then with a concentrated effort from 2009 to 2011, the Institute of Marine Research carried out ecosystem surveys in the Porsanger fjord system as part of the research project EPIGRAPH (see, e.g., Pedersen et al. 2018). The project gathered data on the fjord ecosystem over a number of years, and then modeled impacts of the invasive species red king crab, using Ecopath.

Almost at the same time, through the Fávllis project (UiT- The Arctic University of Norway, 2007–2013), interviews with local fjord fishermen were conducted to capture the LEK of Sámi fjord fishermen on ecological changes in the Porsanger fjord. The Fávllis project collected interviews conducted between 2001 and 2013, and carried out interviews with 27 fishermen and local residents (20 men and 7 women) about their observations of ecological and social changes in the fjord throughout their lifetimes. The interviews were transcribed and assembled in an NVivo database and categorized according to observations of changes in species, social systems, and time periods.

The Fávllis project intention was to integrate LEK with the biological data from EPIGRAPH, assuming that they could be applied in the research and management of the Porsanger fjord ecosystem. The two projects conducted meetings in Porsanger and had discussions about the state of the ecosystem during the project periods. Integration of results from marine science and LEK research, however, turned out to be challenging (Eythórsson and Brattland 2012). Different disciplinary approaches, as well as incompatibility between the long time series of LEK and the shorter time series provided by scientific trawl surveys and samples, as well as challenges with comparable spatial scales for complementary sources like catch statistics, were harder to overcome than first assumed. The challenging experiences from trying to integrate science and LEK raised the question of whether it was at all possible to integrate what were from the outset data from different knowledge systems, produced and performed in what seemed like “different worlds” (ibid., p. 148). Rather than attempting to integrate different forms of knowledge, Eythórsson and Brattland (2012) concluded that what was needed was cross-disciplinary collaboration in partnership with local knowledge producers, such as the Coastal Sami Resource Centre (ibid.). Such co-production approaches are currently supported by the increasing focus on the value of including multiple knowledge systems in biodiversity research (e.g., the Intergovernmental Panel on Biodiversity and Ecosystem Services; Díaz et al. 2015). Rather than attempting to integrate and validate the knowledge systems, the approach recognises that knowledge systems are not necessarily compatible, but that they can still contribute to an enriched evidence base for environmental management (Tengö et al. 2014, 2017). In the field of human adaptation to biodiversity change research, Howard (2013) points to the lack of methods and tools for understanding, documenting and researching human adaptation to biodiversity change. Based on these approaches, this paper is partly a result of continued conversations with marine scientist Knut Sunnanå (formerly the Institute of Marine Research, with a special responsibility for the Porsanger fjord) on the LEK material gathered through the Fávllis project, and the connections between fishermen’s observations and larger-scale ecosystem processes such as ocean temperature and species interactions. Flowing from the conversation between the Fávllis LEK material and Sunnanå’s analysis of the interactions between fishermen’s experiences and fjord ecosystem processes, we produced a social–ecological timeline which could be part of such an enriched evidence base, as well as provide a method for understanding, documenting and researching human adaptation to biodiversity change.

## Constructing a social–ecological timeline for the Porsanger fjord

The basis for the socio-ecological timeline (SET) of the Porsanger fjord is a compilation of characteristic traits for historical periods and “time-constituting events” (or “thresholds of potential concern”), developed according to the fishermen’s perceptions of biodiversity changes in the fjord. Based on previous studies and the Fávllis LEK database, in this paper we use the approach of participant-defined timelines, in order to construct a social–ecological timeline for the Porsanger fjord, with identified major events and tipping points. As opposed to other events, a tipping point is an event that has the effect of completely changing the social–ecological system to the extent that it never returns to the same state that it had prior to the event. Generally, social–ecological history (cf. Ommer 2007) is a history of a social–ecological system (i.e. Berkes et al. 2003), incorporating several different sources, including local knowledge. Identification of temporal scales for social–ecological change, which is the main approach for this study, is generally used as part of participatory rural appraisal methods. Adaptation is generally thought of as actions taken to reduce vulnerability and increase resilience in the face of biodiversity and climate change, which can be taken on multiple scales and in the short and long term (ibid.). Tipping points signal a discontinuity in the history of the system, and to which short- and long-term actions were taken in response to change, such as technological advances or migration (Perry et al. 2011). Andrachuk and Armitage (2015), in a study of the social–ecological transformation of a lagoon fishery in Vietnam from 1985 to the present, identified phases of social–ecological change through participant-defined timelines, focusing on SES elements, interactions, and sources of continuity and novelty. Placing emphasis on socially defined thresholds and “thresholds of potential concern”, they underline that it is a matter of interpretation to empirically know when a tipping point has occurred (ibid.). Similarly, we have analyzed sources of continuity and novelty in the LEK interviews to construct a social–ecological timeline of the Porsanger fjord to identify the importance and effects of changes and response to change over time.

The social–ecological history constructed in this paper is based on major events or that have been addressed in previous literature (Broderstad and Eythórsson 2014) as having dramatic effects on Sami subsistence and livelihood combinations, as well as their responses. The most notable changes are, for instance, overfishing of herring and coastal/fjord cod (*Gadus morhua*), in-migration of harp seals (*Pagophilus groenlandicus*) from the Arctic Ocean from the middle of the 1980s, the introduction of the IVQ system in 1990 that have been singled out by many as an injustice towards Sami small-scale fishermen that marked the end of the traditional Sami fishing livelihood combination (Nilsen 1998; Davis and Jentoft 2001), and the introduction of the red king crab fishery from the early 2000s. These changes are important parts of the social–ecological history as historical or time-constitutive events, and may also be part of or lead up to a tipping point in the social–ecological system. For the purposes of this paper, we see a tipping point as an event that has the effect of changing the social–ecological system to the extent that it never returns to the same state that it had prior to the event.

Broderstad and Eythórsson (2014) identified several events building up to a tipping point in the 1990s for fjord ecosystems in western Finnmark, and point to short- and long-term adaptations among fishermen and communities at the local, regional and national scales. Discussing the resilience and adaptive capacity of the social–ecological system in Porsanger, they argue that the combined effects of the disappearance of coastal cod from spawning sites, depletion of kelp forests and the introduction of red king crab, may be characterized as a *state change* in the ecological system. For the Porsanger fjord, they identified local adaptation strategies during difficult years as: (1) “riding out the storm”, (2) finding alternative occupations, and (3) needing to buy a larger boat in order to fish outside the fjord (ibid.). They also argue that, in the case of Porsanger, adaptive capacity cannot be entirely explained as a trait of the SES itself, but that available adaptive strategies have been dependent upon external (ecological and social) factors on a larger scale, such as the introduction of quotas and other fisheries management measures and financial support for fishing vessels from external sources (Sami Parliament, government) in difficult times. Out of these actions, one might gather that the “riding out the storm” strategy represents the lowest strain on resources while still maintaining fisheries as part of local livelihood combinations. This option has, however, proved to be difficult in Porsanger.

**Fig. 1:**
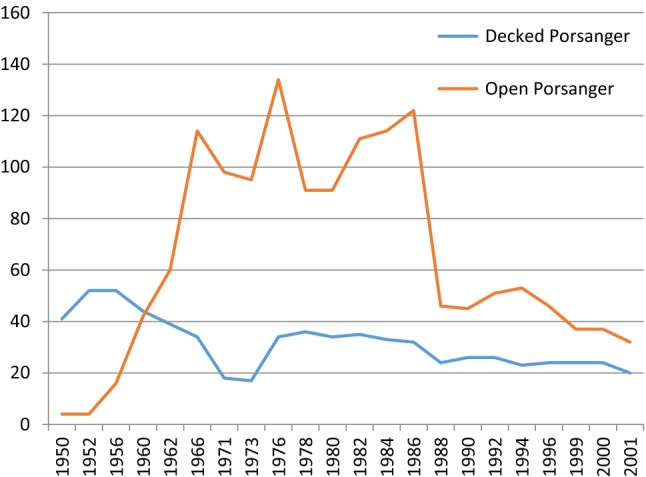
Proportion of decked vessels above 8 m and open, small vessels between 6 to 8 m in Porsanger 1950–2011. *Source* Reproduced from Brattland (2014) with permission from the author

Brattland (2014). in a study of the transition of Porsanger fishermen to an increasingly efficient or “cybernetic” fisheries organization, points at several adaptations in terms of technological change in the fisheries. Fishermen continually adapt to new governance systems and technological systems by incorporating new knowledge, technology and routines into their practices, especially when transitioning to new modes of fisheries organisation (Johnsen et al. 2009). Focusing on the technological development of the local fishing vessels, Brattland (2014) found that fishermen’s adaptations were characterized by ecological intensification on the local fishing grounds in the Porsanger fjord prior to 1989, and an increased spatial range for remaining fishing vessels in the period after the introduction of the vessel quota system. As illustrated in Fig. 1, the number of registered open vessels (typically wooden motorized vessels between 6 to 8 meters of length) increased dramatically between 1950 and 1966 from 4 to 114 vessels, while the number of decked vessels (typically larger vessels with echo-sounders and other fish-finding technology) were reduced. The sharp increase in open vessels from 1950 onwards follows the same development as in the rest of the county, and consisted of new open vessels with outboard engines. In contrast to the rest of Finnmark, however, Porsanger did not experience a decrease in the number of open vessels until the 1988 registry, when it was dramatically reduced by 50% (Fig. 1). The reduction is partly explained by new methods for registering vessels, meaning that many vessels which were not actively participating in the fisheries had been removed, but the main reason is most probably the impact of the seal invasion. During the seal years 1987 and 1988, the number of seals caught in fishing nets on the Finnmark coast increased from 4500 in 1986 to 56 000 in 1987 and 22 000 in 1988. After 1995, the level of seals were back at the level prior to 1986 (between 500 and 2000 each year) (Norwegian Directorate for Fisheries 2004).

For Porsanger, the introduction of the IVQ system in 1990 had a rather minor impact on the number of vessels compared to the drop in 1988, but it made the adaptation strategy of re-entry into the fisheries after the seal invasion was over more difficult. A very low number of fishermen (only 22 out of 79 fishermen) in Porsanger were able to achieve a vessel quota based on the required amount of catch during the years 1987–1989. According to statistics from the Fisheries Directorate, the number of fishermen in Porsanger was reduced by 75% in a matter of years to only around 20, the most dramatic development of all the fjord municipalities in Finnmark (Maurstad 2008). What impact did these changes have on the Porsanger fjord as a social–ecological system?

Throughout the careers and life histories of the men and women who have lived in and subsisted from the marine resources in the Porsanger fjord, environmental change is a central theme. Fishermen are concerned with changes in locations and abundance of cod, saithe, flounder (primarily *Pleuronectes platessa*) and halibut (*Hippoglossus hippoglossus*), but also with the marine habitat and biodiversity such as spawning grounds, kelp beds and sea-bottom conditions, and climatic aspects like wind, currents and ice conditions. Observations of increasing abundance of sea-urchins and decreasing kelp beds which fish fry use to find shelter are also noted as unusual changes by fishermen, especially in the innermost part of the fjord, over several decades. The seal invasion in 1986, however, represents the beginning of the end of a golden period of cod abundance and high participation in the local fishery when re-entry into the fishery was still an available option for many. At that time, both social and ecological events represent abrupt changes in the fjord ecosystem or in society, with great impact upon fishermen’s livelihoods and their ability to adapt. Based on an analysis of both previous studies and the participants’ emphasis on the importance of these changes relative to each other, we have identified two main phases separated by the harp seal invasion, the simultaneous crash in local fisheries, and the introduction of the vessel quota system as a socio-ecological tipping point (1986–1990) from which the fjord never recovered. In the following phase, the king crab invasion started out as a nuisance or “dis-service” (from 2002 onwards) but then developed into one of the most important sources of income for fishermen from approximately 2007 onwards. As indicated in the “Introduction”, there are, however, also challenges with the king crab fishery, which could be compared to the situation preceding the 1990 introduction of the vessel quota system.

Our analytical focus in the following section is on the adaptation actions taken by Porsanger fishermen to cope with and adapt to socio-ecological changes before and after the tipping point, and we also discuss the role of changing ocean temperatures and fishing pressure in the North Atlantic Ocean for fishermen’s adaptations. The goal for the discussion is to arrive at some insights into what actions the Porsanger fjord society can take to mitigate the impacts of climate and biodiversity change in the future, based on experiences with previous change.

## A socio-ecological timeline for the Porsanger fjord

### Phase 1: Traditional adaption of rural fishermen–farmers 1945–1986

The post Second World War period in many respects represents a new beginning for the areas that were burnt down during the German scorched earth strategy, which makes it a natural place to start the social–ecological timeline. This is in general a period characterized by increasing prosperity and a relatively healthy marine ecosystem. The traditional adaptation of the rural fisher-farmers of Porsanger (and northern Norway in general) of combining fishing with other sources of income in this period could perhaps be characterized as “autochthonous” (Howard 2013). Especially, the 1960s and 1970s were a period when the fjord fishery was one of the most important livelihoods for most households in Porsanger. It kept fish buyers busy, and cod stocks were still healthy and able to provide both dinner on the table and monetary income. Due to economic development and investments in fishing vessels and the fishing industry, conflicts between traditional fjord fisheries and commercial fisheries soon made themselves felt. In the 1950s and early 1960s, large quantities of herring, mostly juvenile, gathered in Porsanger (Pettersson 1994) where they were fished by a large fleet of effective, non-local purse seine vessels. Large saithe were abundant in the 1950s (summer–autumn) but diminished during the 1960s and 1970s, due to increased fishery by industrial fishing vessels (Andersen and Persen 2011: 71). The most dramatic events noted by fishermen are the sudden collapses of the herring fishery and the saithe fishery due to overfishing by seine vessels.

Cod was abundant during the 1970s: five local delivery stations operated in the Porsanger fjord and the considerable amounts fished in Porsanger were delivered to stations outside the fjord. In 1972, a fish-processing plant was established in Billefjord by Olav Bull from Repvåg, to which the fishermen in the inner part of the fjord took their fish:

We all fished – people bought boats and – those years people bought boats and skimmed the cream. Those were the 1970s when there was a lot of fish in here, and in the beginning of the 1980s, before the seal problems started. In the winter the larger vessels fished in Olderfjord – in the net season. But we were many of the smaller boats that fished just in here. But since the last five or six years [2003] it has been the way that in July and August, the fish disappears from here.

However, increasing conflicts on the cod spawning grounds between conventional gear (gillnets, mainly local vessels) and active gear types (Danish seine, mainly non-local vessels) followed. As catches started to diminish, fishermen changed their gear to adapt.

Afterwards I regret that we used small-meshed nets in the Porsanger fjord, the amount of kilos caught decreased the more we fished, and in the end there were rules for the mesh size. In the beginning we controlled the mesh size ourselves until the rules came later. Then the fishermen talked about the catch getting smaller, but we had ourselves to blame. And after a year of restrictions the catches were better. The size went up, but we caught less fish. I think they should have done more to preserve the fish in the fjord. I will way that we have been part of the destruction. When in addition boats from the nearby areas came with sink nets the ocean was emptied. This was in the middle of the 1970s.

Gillnets were modified for deeper water and with reduced aperture to catch smaller sizes, and the change from cotton and hemp to synthetic fibre also made gillnets more effective. Some local fishermen foresaw an impending catastrophe based on the intensity of fishing on their traditional fishing grounds.

There were so many nets there that (..) it was like a cloudberry field. It was orange with floats. And it was the spawning fish they took, or we took, I was a part of it too. But as 16-year-old (in the 1970s) I said to the fishermen that this has to end, you cannot take the spawning fish. Some day it will be empty. And it turned out, that when you take the spawning fish…. Then no new fish. Now you don’t see a fishing boat there anymore.

As local fishermen worried about the situation, the local fishermen’s association asked for protective measures. This had political repercussions as the regional fishermen’s association would not close off the rich fjord fisheries to their members. In essence, the coastal Sami uprising (Nilsen 2003) was a conflict between local Sámi fishermen and the Finnmark fishermen’s Association, who decided to exclude two members from Porsanger because they had raised protection of local spawning grounds as a Sámi rights issue (Eythórsson 2008). As a result, fisheries regulations that prohibited Danish seine fishing in the spawning season became effective for most spawning sites in Porsanger fjord during the 1980s. This did not, however, prevent some spawning grounds to be fished down. In a study by Maurstad and Sundet (1998), two of the previous productive spawning grounds in Porsanger were declared “dead” as a result of overfishing, in Olderfjord and Billefjord.

While overfishing was high on the political agenda at the time, fishermen also had hypothesises about the role of environmental change. A fisherman born in the 1930s started noticing changes from around 1975: that the sea urchins living on the bottom began multiplying and the sea bottom changed. He also noted the increasing ocean temperature from the middle of the 1980s:

It is climate change. Everything used to freeze, you had to go as far as Bevkop or even further to see open water, when you came to March. But now, when you go out here you can see the sea, open sea

This coincided with spring coming earlier. In the late 1980s, fishermen noticed that kelp was disappearing in the fjord, the apparent reason being an explosive increase in the number of kelp-eating sea-urchins (supported by Sivertsen 2006). Sivertsen and Bjørge (2015), who studied the process in the Porsanger fjord, found that sublittoral macroalgae had been subject to downgrazing by sea urchins to such an extent that it had left barren grounds in some localities in the outer and middle fjord. One local hypothesis was that the natural enemies of sea-urchins were depleted. Some had a theory that the population of Atlantic wolffish (*Anarhichas lupus*), known to feed on sea-urchins, had diminished. Wolffish are not targeted by fishermen, but the otter (*Lutra lutra*) is known to be an able wolffish-hunter. Whereas traditionally otters were hunted and sought after for their fur, it is now a protected species and growing in numbers. Disregarding the causal relationship, locals do not have the opportunity to hunt otters to facilitate regrowth of kelp beds.

**Fig. 2:**
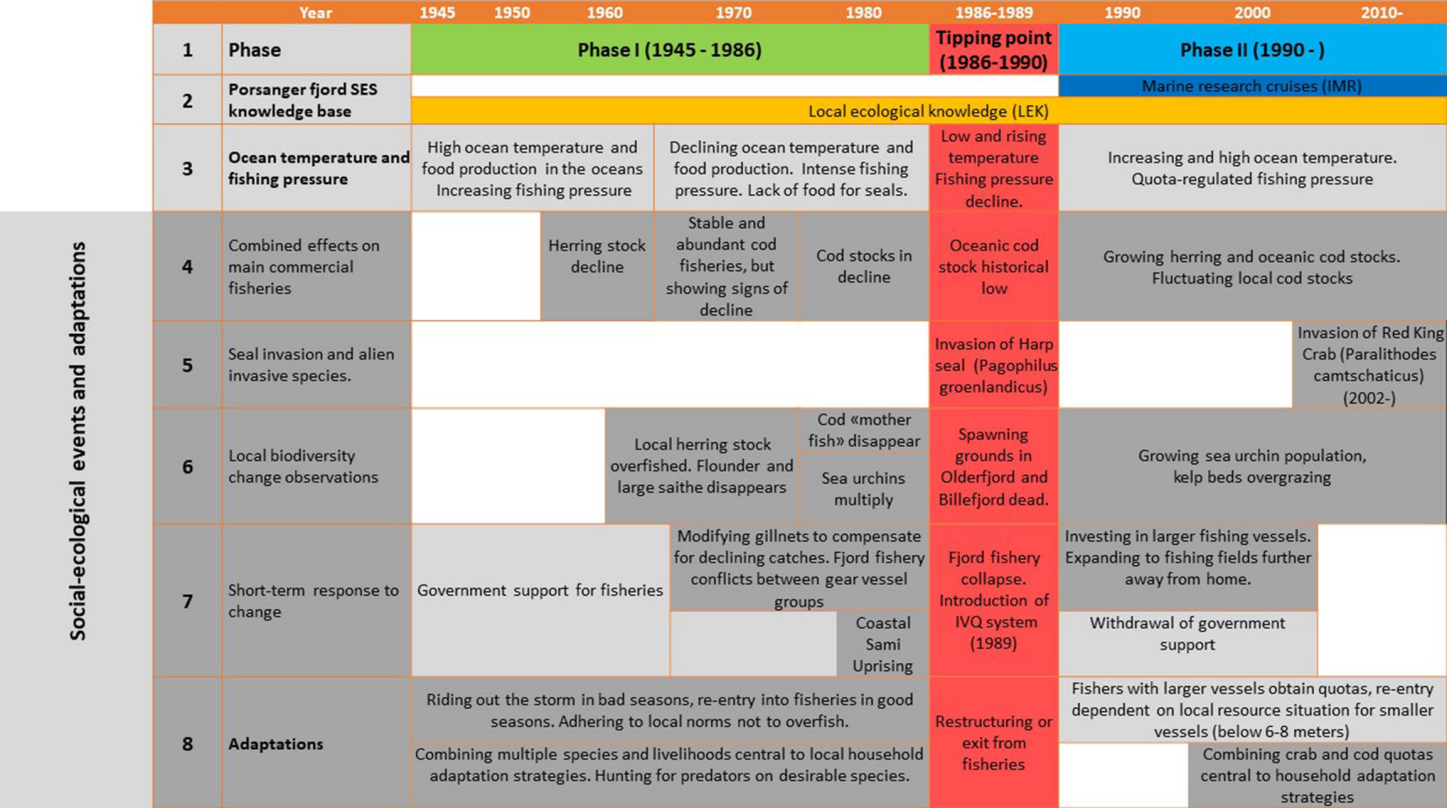
Social–ecological timeline for the Porsanger fjord with participant-defined phases and tipping point (1), temporal extent of LEK and marine science (2) and changes in ocean temperature and fishing pressure (3) relative to social-ecological events and adaptations (4–8)

According to Sutton and Hodson (2005), ocean temperature declined from the very warm years in late 1930s and 1940s through the 1950s and 1960s to a minimum in the late 1970s (see Fig. 2). This is also related to the decline in the herring stock during the same period. Herring are assumed to respond directly to lack of food, and temperature is assumed to be a good proxy for primary and secondary production in the oceans. The supply of zooplankton (secondary production)—both in the open sea and in the fjords—directly influences the abundance of herring (Sunnanå, personal communication). What fishermen saw as a direct consequence of overfishing on herring and saithe in the 1960s and 1970s might then also be related to the declining production of zooplankton, which is difficult to observe by the human eye. In the 1970s and 1980s, the ocean temperature was low (although fluctuating), which probably also resulted in a low production of food in the ocean as well as in the fjords. In the 1980s, recruitment improved for both herring and cod. This probably led to heavy predation on zooplankton in the open sea, as well as heavy predation on small capelin, causing a reduction of the capelin stock and thus lack of food for cod. This again led to a shortage of food for seals, which sent them on a hunt for food to the Norwegian coastline.

### The tipping point: Invasion of seals, local cod collapse and introduction of IVQ system (1986–1989)

According to Nilsen et al. (1992), a consequence of the low ocean temperature and the collapse of the capelin stock in the 1980s was a severe food shortage for marine mammals in the Arctic Ocean, particularly seals that feed on capelin and small crustaceans. As the food sources diminished in the open seas, numbers of seals moved closer to the coast and into the fjords of Finnmark and Troms counties. Large numbers of seals are known to chase away fish in areas where they appear, and they also get entangled in gill nets when they try to feed on fish caught in them (Nilssen and Haug 1995). Seals are also hosts for parasites (*Anisakiasis simplex*) on cod that reduces the quality of the fish. In 1979, harp seals in large numbers appeared in eastern Finnmark. In the following years, the seals spread westwards and reached Porsanger in late 1986, where the cod fishery crashed.

The LEK narratives are rich in information about the period leading up to the dramatic seal invasion, and the following impacts on cod, saithe, and flounder fisheries.

The fish disappeared before Christmas in 1986, from the whole fjord, until early summer 1989. The seals were all over the place. Gillnets filled with seals and destroyed. The bigger fishing boats went outside the fjord to fish, the fjord fishermen with small boats were forced to quit fishing.

The number of seals in the Porsanger fjord was at its highest in 1987–1988 and the local cod fishery collapsed in 1987, with a reduction of the fishing fleet by 50% (Brattland 2014). The owner of a local delivery station noted the impact on catches to the fish receiving stations in the fjord:

Before the collapse, the deliveries were between 1,5 and 2 million kilograms of cod, from the west side of the fjord (Olderfjord, Smørfjord and Repvåg). The catches from Smørfjord alone were between 500.000 and 700.000 kilograms. After the collapse, it went down to about 90.000 kilograms.

The cod did not return to the spawning sites in the inner part of the Porsanger fjord after the seal invasion, but continued to some degree in the outer part of the Porsanger fjord. Local fishermen had a theory that the cod were scared off to deeper waters outside the area of local gear restrictions that were in effect for the spawning areas in the fjord. As the fish gathered outside the restricted areas, they could easily be caught by larger vessels using Danish seines and long lines. As a result of the declining fishery, the fishermen with the smallest vessels which were not equipped to follow fish over longer distances, dropped out of the registries (Brattland 2014). Many of these vessels never re-entered the fishery after the introduction of the IVQ system in 1990, even though some may have had the intention to enter the fishery again after the storm was over.

Under these conditions, combined with overfishing of the cod stock, the fisheries managers struggled to avoid what they saw as a cod collapse in the late 1980s. The total allowable catch for cod was strongly reduced in 1990, and IVQs, already in effect for the offshore fisheries, were also introduced in the coastal fishing fleet (Christensen 2017). For the Porsanger fishermen, the collapse in the fisheries had, however, already occurred in 1986, thus making it hard to accept the link between local overfishing and vessel quotas at the time. The introduction of vessel quotas for the small-scale fishing-fleet in 1990 represented a limitation of catches and exclusion of fishermen who did not meet the requirements for quota allocation in terms of their vessels’ catch record for the last 3 years. Since many of the Porsanger fishermen had been unable to maintain their fishery during the seal years, they did not meet the requirements for allocation. Many small-scale fishermen quit fishing and never returned to fisheries as they had found other occupations (Broderstad and Eythórsson 2014).

### Phase 2: Adapting to the new system and arrival of the Red king crab (1990–2010)

The quota system revealed a tension between traditional ways of limiting catches and the new way of thinking:

Even though I have a quota that is 10 or 15, or 20 or 100 tons for that matter, I fish as much that I decide that «I don’t need to fish anymore, it is enough”. (..)I mean, before this quota system was introduced in 1990, when we didn’t have a quota. Then we fished until the season was over, and we quit when it was bad. And didn’t care to fish more. After the quotas you don’t hear anything else than that you need to fish up your quota. And that is even if it is 10 or 100 or 150 tons. That is, you need to fish up that quota.

The new system had consequences for the adaptation strategy of combining livelihoods to support household economies. In order to catch their allotted quota with their vessels and keep up the fisheries activity, and thereby the right to stay in the fisheries, the fishermen who remained and obtained a quota adapted to the new system by investing in more effective and mobile fishing vessels that could fish farther away from home. This meant, for instance, narrowing the range of livelihood combinations to invest more time and resources in fisheries, and stationing vessels in communities closer to the richer coastal fishing fields in the main cod fishery season (Brattland 2014). Decommissioning of vessels was also an action used by the government to get rid of overcapacity and facilitate participation in the closed quota-regulated fisheries. Instead of local norms and values guiding when one had fished enough, the quota decided when it was time to stop fishing. The quota system thus spurred responses on the personal and household levels; it represented a break with previous norms and societal mechanisms; it changed with which vessels and where active fishermen fished; and not least it led to political change. Most importantly, the newly established Sami Parliament used the opportunity to argue that coastal Sami had been hit hardest by the new regulations, since so few fishermen in coastal Sami fjords had acquired a quota due to the seal years prior to the introduction of the system (Broderstad and Eythórsson 2014). Some compensatory measures were introduced a few years after the quota system was in place, such as economic support from the Sami Parliament to cover loans on fishing vessels (the price of which includes the value of the quota). In general, the new system radically changed the traditional livelihood adaptation all along the Norwegian coast.

In the northeast Atlantic, herring and cod stocks grew rapidly as the ocean temperature rose in the 1990s. The capelin stock suffered from this increase, and only in periods when the abundance of young herring was low in the open sea would the capelin stock recovered temporarily. Combined with the increasing but fluctuating ocean temperature in the 1980s, these conditions may have resulted in a fluctuating cod stock, in the open sea as well as in the fjords (Sunnanå, personal communication). Around 2010, ocean temperatures reached the same high level as in the late 1930s and early 1940s. Simultaneously, the herring stock reached biomass levels comparable to the levels in the 1950s, and the size of the mature stock of oceanic cod the same levels as in the 1950s. However, the coastal cod stocks have not recovered to the same degree as the oceanic cod. Adding to the meagre recovery of cod fisheries in the fjord,^1^ and fishermen’s concern for other species like flounder that continue to be absent, the present social–ecological system is in a very different state. This might indicate that high ocean temperatures have different impacts on local and oceanic cod stocks.

Around the turn of the century, the red king crab reached Porsanger. The arrival of king crab was the result of transport of living crab from the Pacific by Russian biologists to the Barents Sea in the 1960s (Pinchukov and Sundet 2011). The red king crab has gradually expanded its territory and has shown up in increasing numbers in Porsanger since 2000. At first, it was only a nuisance, especially for gillnet fishermen, but after 2002, local fishermen have been allowed to participate in a commercial and increasingly lucrative crab fishery. It grew to become the main fishery in the fjord in terms of catches and income. Due to the amount of crabs caught up in gillnets in the inner part of the fjord, fishermen were, however, forced to relocate their vessels to fishing grounds further away from their homes.

After a couple of years, I moved to another fishing area. After 2002, I left the fjord almost for good, I have been here only sporadically. I have been fishing on the western side, from Kokelv and further off the shore, and from Havøysund and towards Repparfjord. But it was because of the arrival of the crab that I moved, it was not possible to fish with gillnets.

From 2008, the fisheries authorities opened up a so-called “extinction fishery” in the inner part of the fjord, aimed at decimating and halt the spread of the rapidly expanding stock of king crabs. Although the decision was controversial among fishermen, the fishery boomed to such an extent that the remaining receiving station in Smørfjord had trouble receiving all of the catch.

When the stock was down to a sustainable level, the fishery was closed again in 2015 and was limited to those with vessels below 6 m and who already participated in other fisheries. The crab is currently a valuable commercial species, and the catch regulations are favorable for most participants in the fishery. For the remaining active fishermen, they can stay in waters closer to home, not having to move between fishing grounds towards the coast to catch the cod and crab quotas throughout the yearly cycle. Paradoxically, the new fishery thus allowed some fishermen to station their vessels close to home as resources have become abundant again. For others, such as fishermen with vessels below 6 m, this meant that the option of including the new species in their livelihood system was cut off, and investing to enter the closed fishery or finding alternative occupations to combine with non-quota regulated fisheries were the remaining options. For this group of fishermen, king crab will provide more disadvantages in terms of destruction of fishing gear than economic advantages or opportunities to continue local livelihood combinations.

According to Pedersen et al. (2018), the king crab does not seem to have a significant effect on the cod stocks. The long-term ecological impact of the crab is, however, not known (Sundet 2008), and fishermen worry that it is yet another contribution to the degradation of the fjord ecosystem. Scientists at the EPIGRAPH project collected samples using different types of gear over a 4-year period at several stations in the fjord, aimed at analyzing the fjord’s ecological processes as a top–down system driven by predators (Pedersen et al. 2018). The fjord was modeled using Ecopath with Ecosim (EwE), analyzing the impact of the red king crab on invertebrates, mainly concluding that the ecosystem in Porsanger is relatively resilient in face of the king crab invasion, and that cod fisheries remain relatively undisturbed.

Based on ecosystem model simulations run by the EPIGRAPH project, however, a likely scenario is that king crab will deplete sea-urchins, thus contributing to increased regrowth of the kelp forests that are important nursery areas for cod (Pedersen et al. 2018).

## Discussion

As the SET that we have constructed for this paper shows, the SES before and after the tipping point (1986–1990) can be characterized as almost two completely different systems, also in terms of adaptation options. During the first phase, local and short-term adaptations to dwindling resources included changing mesh sizes in fishing gear, adhering to local norms and values not to overfish both within local fishermen’s groups, as well as political organization against overfishing by encroaching vessel groups (the Coastal Sami Uprising). Entering fisheries and establishing receiving stations to connect fishermen with the market was supported by government incentives, and it was relatively easy to re-enter fisheries at a later time if the season was bad. Locals also referred to previous adaptation practices such as hunting for predators (otters and seals) to balance out pressures on important (cod) and vulnerable species (i.e. the sea-urchin–kelp connection). Active fishermen in Porsanger today may refer to the herring collapse in the 1960s as an important historical event which was massive, but it did not change the entire social–ecological system of the fjord. There was thus little need to look for other occupations at that time, when other fisheries were still available within the Porsanger SES.

Some fishermen warned of the consequences if fishing continued at the same rate that they saw happening in the mid-1980s, and the rapid expansion of the fishery led to protests from the Sami fishermen. There were informal mechanisms in place to avoid overfishing such as norms against catching “mother fish” and, as expressed by one of the fishermen, a value to not fish more than he had need for. These norms, or institutions (sensu Ostrom 2015), can indeed be interpreted as a key aspect of the autochthonous adaptation of a rural society to the threat of unsustainable resource use (see for instance Nilsen 1998). In the interviews conducted by the Fávllis project, fishermen do express worries about the impact of active gear types, but they also readily admit that they themselves had a part in depleting the local cod stocks by taking the spawning codfish and changing the mesh sized in their gillnets. In our opinion, this illustrates that rural societies, or any society, are comprised of a diversity of complex actions of adaptation and mal-adaptation. In most cases these adaptations are the products of very particular interactions between social, technological and ecological factors which are difficult to conflate and represent under the label of a single adaptation strategy independent of a longer historical timeline. Adaptations at the local scale will, however, become easier to see once they are investigated in their historical, social and ecological context, as we have done in the form of a SET.

Fishermen’s own explanations emphasize the role of gear conflicts and overfishing of stocks as the cause of resource decline and environmental degradation. One of the limitations of the LEK is that it is limited to the experience range of fishermen, thus missing the role of larger-scale both social and ecological processes and their impacts. To understand larger-scale processes impacting local adaptations, we need to take into account larger-scale social–ecological and environmental processes.

### Adapting to societal change

In the first phase, the social drivers behind the increased fishing effort in the period after World War II was an enormous government intervention to transform local household economies. The period was in general characterized by government incentives to support fisheries as one of the most important primary industry occupations in the country. With an active state policy for protecting fishermen’s incomes through the Raw Fish Act^2^ (1938) and the Main Agreement (1964), fish prices were stabilized and provided labor opportunities to a population sorely in need of cash to rebuild the country. The traditional rural fisher–farmer adaptation with a combination of subsistence and commercial fishing with small-scale farming (Eythorsson 1993; Nilsen 1998) also changed when welfare increased. Industrialization and investments in technological development of the fishing fleet, and the establishment of several fish-landing stations, contributed to a growing number of more effective fishermen and vessels both local and foreign, which increased the fishing pressure on vulnerable stocks of herring, saithe and cod.

The first social–ecological phase was coming to an end when the seal invasion and the introduction of the IVQ system occurred at the end of the 1980s. The number of fishing vessels had dropped drastically (see Fig. 1), leaving fewer fishermen and fishermen’s families to potentially enter the fisheries again once the strategy of “riding out the storm” or finding alternative occupations during difficult years (Broderstad and Eythórsson 2014) had been abandoned. During the following phase, adapting to the quota system became the main option for the remaining fishermen, which meant restructuring their livelihood combination to invest in fisheries as a main occupation. This again had implications for where fishermen fished (moving further towards the coast from other fishing ports instead of local ports), and those fishermen who qualified also then had the opportunity to participate in the increasingly lucrative king crab fishery. The new system, however, created not only winners but also losers, in the sense that fishermen with low activity were unable to flexibly incorporate species (cod, king crab) into their livelihood combinations.

By the 1990s, the state increasingly withdrew its support to facilitate the introduction of a liberal market economy (light grey fields in row 7, Fig. 2). The introduction of the quota system was thus not only motivated to preserve a sustainable cod stock but also to decrease the number of fishermen and vessels engaged in the fishery to increase profit for the remaining active fishermen. A major focus of the fisheries and the historical literature of the region has been the injustice done to many small-scale fishermen and coastal communities upon the closing of the coastal commons and the introduction of the IVQ system in 1990 (Maurstad 1997; Hersoug 2005) investigated the tension between internal norms as a guide for local fishermen’s resource management practices and its incompatibility with the new quota system.

It is perhaps surprising that the invasion of the seals to the Porsanger fjord marks a tipping point for the SES, since the introduction of the IVQ system has received most of the attention in northern Norwegian history as the single most dramatic event that closed the previously open coastal commons in northern Norway (Hersoug 2005; Christensen 2017). For the Porsanger fishermen, however, the seal invasion had already caused a dramatic cut in the number of fishing vessels in Porsanger before the IVQ system was introduced (Brattland 2014). The number of larger vessels registered in Porsanger stayed relatively stable through the tipping point around 1990, since the bulk of fishermen had already disappeared with the seal invasion (Brattland 2014). For the ecological system, the seal invasion was fatal for local cod stocks, and represents a break in the social–ecological history of the Porsanger fjord. The new quota system had implications for adaptation options, which again may influence the extent to which communities are prepared to plan for climate change and transition to a sustainable future.

The arrival of the red king crab first represented an ecosystem dis-service to the fishermen in the form of destruction of gillnets in the small, but slowly recovering, cod fishery. According to the Shackleton et al. (2007) framework, the red king crab can be characterised as a “useful, aggressive species” with benefits exceeding the costs for the rural population after the introduction of quotas to the cod fishery and upon establishment of lucrative market relationships. For the biodiversity of the fjords, however, the red king crab has a quite destructive effect on bottom fauna, but the fisheries seem not to be affected as severely (Sundet 2008). In fact, according to Pedersen et al. (2018), the king crab may actually contribute to regrowth of macroalgae, as red king crab is a major predator on sea-urchins.

For fishermen in Eastern Finnmark and in Porsanger, the red king crab quota represents a new beginning as the cod fishery alone was not able to provide enough income for households in the communities after its decline. This option is, however, only currently available to those with vessels above 6 m. Since the introduction of the red king crab, the SESs in parts of the Finnmark coast have been transformed as a result of adaptive strategies employed both by the local population and local governments. While these strategies were initiated locally, they were made possible by environmental change (introduction of the red king crab fishery and recovery of the cod stocks) as well as strategies at a larger governance scale (changes in fisheries regulations and political and economic support from the authorities).

### Adapting to environmental change

Environmental change is linked to, and in some cases explained by, changes in human actions such as improved fishing technology (increased efficiency of gillnets and Danish seine), fisheries regulations (quota regulations and gear restriction on spawning sites, etc.), and market change (loss of market for seals, new market for king crab). Some of the changes in the ecosystem, however, are not linked to specific human actions by the fishermen, like the increase in the population of sea-urchins, the seal invasion in 1987–1989, and the introduction of the kind crab. Marine scientists may explain these changes as caused by changes in ocean temperature and large fluctuations in key stocks in the system (Toresen and Østvedt 2000; Jakobsen and Ozhigin 2011). The decline of kelp beds, the invasion of seals and the introduction of king crab are examples of changes to which local fishermen had no legal adaptation strategies. The local government, however, actively searched for answers to the puzzle of the “empty” Porsanger fjord beyond the role of overfishing, and sought the assistance of the Institute of Marine Research and the University of Tromsø through the Fávllis project. Large-scale climate and biodiversity changes such as rising ocean temperature and the collapse of the capelin stock were beyond the observation capability of local fishermen, yet these factors had a real impact on the Porsanger SES and on key ecosystem services.

The impact of changing ocean temperatures is, however, difficult to identify for the fjord fisheries. Low temperatures in the 1970s and 1980s resulted in a lack of primary and secondary food production in the oceans and thus led to fluctuating cod stocks. The disappearance of the kelp forests in the fjords may be caused by large climate fluctuations that have changed the balance and relationships between different fish stocks occupying the fjord systems. The lack of large predators, such as cod, in the fjords may be due to lack of food, e.g., herring. Fewer large predators may again give room for other predators, i.e., sea-urchins, that feed on the kelp forests to be numerous, as cod may be one of the predators on sea-urchins (Sunnanå, personal communication). These links between ocean temperature and impacts at the local scale are however difficult to identify, and in need of more research attention.

## Conclusion

What adaptation strategies should be facilitated in order to maintain the resilience of coastal social–ecological systems? Based on the discussion above, it seems clear that adaptation options for coastal Sami fishermen vary with the state of the ecosystem and with the constraints and possibilities offered by management systems. What the Porsanger SET illustrates is that there was never a time when ecological conditions were stable, as ecological fluctuations seem to be inherent to the fjord ecosystem, as they are in many Arctic ecosystems. In terms of adaptations, the option of entering and re-entering the fishery as part of a flexible rural livelihood approach, however, seems to be constrained by the introduction of the vessel quota system in 1990. Even though opportunities to incorporate king crab fishery as part of traditional adaptations improved between 2004 and 2014, new rules seemed to create difficulties for the smallest group of fishermen, who are most likely to be combining fishing with other livelihoods. This may decrease the capacity to cope with further changes such as increased ocean temperatures and thus even more unpredictable ecological conditions in the future. In the current context of climate change, it seems important to facilitate long-term, sustainable adaptations and to initiate actions to mitigate climate change. What may such actions look like?

Based on the ability of fishermen to cope with change through diversification of economies in the past, an option could be to facilitate (re-)entry into fisheries as part of flexible livelihood combinations. This could potentially foster environmentally sustainable fishing practices in a future diverse and green economy. Increased exploitation of adjacent resources by fishing vessels that are not dependent on oil or diesel could also represent a viable strategy to cut climate gas emissions while still maintaining sustainable livelihoods among the rural population. The current management system, however, favors effective vessels and fishermen with large capture capacity and spatial mobility. This may, however, also change, for instance in a future where market conditions no longer facilitate fossile fuel-driven fisheries. What will happen in such an event has already been suggested by how decommissioning of superfluous vessels to deal with over capacity puts new and efficient vessels out of business (Brattland 2014). With the increasing urgency of transitioning into a new and green economy driven by climate change, investing in electrified vessels could be a relevant adaptation option for municipalities like Porsanger.

The interactions between ocean temperature and its different implications for oceanic and fjord ecosystems are complex and difficult to predict. The way these changes have been experienced are, however, more accessible through the memories and traditional ecological knowledge of fishermen. In their experience, the disappearance of kelp beds was something they were not used to, in an ecosystem where fluctuating fish stocks and invading species were part of their daily lives. As indicated by a report by the Nordic Council of Ministers (2017), the supporting ecosystem services of kelp forests is important for ecosystem health, as macroalgae are important for storing and capturing of carbon in the oceans. The local marine research station in Porsanger has for years experimented with regrowth of macroalgae in the fjord (Sunnset 2010). From a climate mitigation perspective, restoration and culturation of kelp beds is thus an action that could be planned into the coastal zone plans for the Porsanger fjord.

In terms of research on human adaptation to climate and biodiversity change, the way we have constructed the work around the social–ecological timeline for Porsanger brings attention to the relationship between local observers and science. When science has few conclusive answers for the causes of environmental change, traditional and local ecological knowledge on what a healthy ecosystem should look like could thus provide guidance on what adaptive actions could be taken. The experiences and responses of locals to not only changes in abundance of certain species but also to biodiversity change in general is a good starting point for analyzing changes in ecosystem functions. Monitoring of local biodiversity changes such as those occurring in remote rural societies is, however, still not a focus of research, nor are there any programs to initiate monitoring of biodiversity change in these societies. Regarding the involvement of local fishermen in future monitoring and research on the Porsanger fjord, it is notable that no fishermen input was used in the EPIGRAPH project, which modeled the ecosystem using Ecopath. In a study using EwE, Bevilacqua et al. (2016) argues the potential of FEK to fill in knowledge gaps of in ecosystem modelling using Ecopath, especially in data-poor situations. This is where partnerships with local knowledge producers in Porsanger could constitute a difference for monitoring of the state of the changing ecosystem using traditional and local ecological knowledge of the past ecosystem as a reference point.

This should not only involve local knowledge holders (Davis and Ruddle 2010; Stephenson et al. 2016), but ideally be conducted in collaboration with science, to foster both adaptive capacity and self-governance, not only in research centers but also in rural communities (Colin-Castillo and Woodward 2015). Such a monitoring system would need to define a set of indicators for biodiversity change, which could be participant-defined based on the construction of SETs. If ecological indicators were co-produced and made explicit by fishermen and biologists, monitoring could then be a task for the fishermen as part of a citizen science or community-based monitoring programme (Tengö et al. 2014). In addition, local participation in the gathering of and reflection upon this information can also contribute to greater awareness of climate and biodiversity change in local historical processes, and generate source material for environmental history (Weines 2016). Keiner (2013) and Taylor (2013) reflect on the future lack of source material about ecological changes and methodological challenges in extracting LEK from old sources. They conclude that a focus on preservation of LEK in the present can be an appropriate response. Local observers could participate through citizen science contributions to environmental monitoring using smart devices. Whereas local observations can be instantly interpreted and communicated to others, scientists first need to gather data, analyze them, have them reviewed, and only then communicate findings. The traditional way of producing and processing scientific knowledge is often too slow for the quick and fluid flow of current environmental web monitoring systems. In the Porsanger fjord, a local indigenous organisation has already built an online database where local narratives and observations on biodiversity change are increasingly posted and discussed (Andersen and Persen 2011; CSRC 2017). Building on LEK, as we have done in this paper, incorporating marine science results, and then co-producing knowledge of large-scale biodiversity change to build time-series for the future, could provide a database of explanations for and indicators of biodiversity change. Last but not least, our work documents the adaptation actions taken by locals, government and science when faced with biodiversity change. This article is in itself an action in response to change, as it would not have been written had not the Porsanger municipal local government sought to bring their fjord “back to life” by reaching out to researchers in an attempt to understand and mitigate the effects of climate and biodiversity change.

## Footnotes

1 In the winter season of 2011, there were, however, reasonable catches of cod in the fjord, for the first time in more than 20 years.

2 The Raw Fish Act established a monopoly for co-operative fishermen’s organizations which negotiate prices with fish-buyers on behalf of the fishermen.
